# Editorial: Responses to Climate Change in the Cold Biomes

**DOI:** 10.3389/fpls.2019.00347

**Published:** 2019-03-28

**Authors:** Hans J. De Boeck, Erika Hiltbrunner, Anke Jentsch, Vigdis Vandvik

**Affiliations:** ^1^Department of Biology, Centre of Excellence PLECO (Plants and Ecosystems), Universiteit Antwerpen, Wilrijk, Belgium; ^2^Department of Environmental Sciences, Institute of Botany, University of Basel, Basel, Switzerland; ^3^Department of Disturbance Ecology and Vegetation Dynamics, Bayreuth Centre of Ecology and Environmental Research (BayCEER), University of Bayreuth, Bayreuth, Germany; ^4^Department of Biological Sciences and Bjerknes Centre for Climate Research, University of Bergen, Bergen, Norway

**Keywords:** climate change, alpine, arctic, invasive species, biodiversity, climate extreme, range limits, Antarctic

Climate change is a global phenomenon, with particularly rapid changes observed in many mountain ranges, the arctic, and the Antarctic Peninsula, where temperatures have risen at twice the global average or more (Gobiet et al., 2014; Johannessen et al., 2016). The accelerated warming in these cold biomes is mainly due to various positive feedbacks, such as albedo effects related to declining snow and ice cover (Screen and Simmonds, 2010; Pepin et al., 2015). Apart from temperature, precipitation patterns have been changing in most of these regions as well, with varying impacts on snow pack depths, growing season length and in some regions also on the prevalence of summer drought (Gobiet et al., 2014; Bintanja, 2018). While these rapid environmental changes have the potential to significantly alter responses and dynamics of the local ecosystems (e.g., Niittynen et al., 2018), logistic challenges have constrained research in these often remote locations. In this research topic, we collected a number of studies that have overcome these challenges to report on the responses of cold biome ecosystems to various aspects of climate change.

In trying to predict how ecosystems will respond to rapid climate warming, insights may be gained from warm periods in the past. Tree rings provide important information on historic temperatures at the treeline, where the primary limiting factor for tree growth is considered to be growing season temperature (Paulsen and Körner, 2014). In this research topic, Lange et al. investigated possible distortions of temperature-growth relationships along a transect running from Finland into Western Siberia, and found that the temperature signal of *Pinus sylvestris* differed markedly between climate regimes. This could challenge the assumption that the correlation between temperature and radial tree growth limitation is stable over time. The micro-site climate is likely to play an important role here (Körner et al., 2016), which has implications not only for temperature reconstructions, but also for other applications where absolute growth is important, such as dynamic vegetation models.

Experiments are powerful tools for establishing cause-effect relationships and testing specific hypotheses regarding climate change impacts, but researchers should be aware of the limitations of different methods and techniques (e.g., De Boeck and Nijs, 2017). Yang et al. combined three warming approaches in a single study in the Hengduan Mountains (China) to establish their influence on indirect (biotic) climate effects. They concluded that differences exist especially regarding the rate of colonization by new species, which is more rapid in whole-community transplantation studies compared to experiments employing open top chambers (OTCs). Yang et al. suggest that combined approaches therefore provide opportunities in disentangling direct effects of warming from indirect effects mediated through novel plant-plant interactions.

Sierra-Almeida et al. used OTCs in the first *in situ* study of the impact of climate warming on the freezing tolerance of the only two naturally occurring vascular species in maritime Antarctica (*Colobanthus quitensis* and *Deschampsia antarctica*). Their results show that temperature increases can lead to a reduction in freezing tolerance, highlighting the complexity of warming effects and the importance of phenotypic plasticity in coping with climate change. In a transplantation experiment, Henn et al. demonstrated that leaf trait plasticity enabled alpine plants to persist within communities exposed to warming. This encompassed mostly convergence toward optima found in the local, pre-adapted plants when transplants were moved to warmer conditions. The authors note that the extent and importance of trait plasticity is species-specific, as was also reported in the study of Cui et al. who found low plasticity in leaf area of *Viola biflora var. rockiana*. For this species, the key to survival under climate warming seems to hinge on being able to avoid competitive effects from the surrounding community. Both studies demonstrate that climate change is likely to lead to range contraction and extinction especially for those Himalayan alpine species that are unable to adjust important traits, unless competitive exclusion is avoided by management intervention (e.g., mowing or grazing). The declining species richness in response to warming observed by Yang et al. is a further illustration of the risk of biodiversity loss in mountain ecosystems, with alpine-restricted species seemingly more sensitive than wide-ranging species according to Winkler et al.

While uncovering the mechanisms that determine plant distribution ranges along elevational gradients is highly relevant, fewer studies exist on latitudinal range limits. Liu et al. investigated the variation in non-structural carbohydrates and nutrients in *Quercus variabilis* along a 1,500 km north-south transect in China. They conclude that carbon or nutrient availability are likely not the main driving factors of range limits of this species, while recruitment limitation seems more important at the species' northern edge (Gao et al., 2017). Species ranges are affected not only by climate, but also by direct human intervention. Vetter et al. looked into the effects of *Lupinus nootkatensis*, a high-latitude invader introduced in Iceland in the 1940s, on the native flora. The invasive legume tended to decrease plant species richness in the most species-rich habitat (heathland), while promoting especially late-successional and ruderal species in woodland and grassland. As the habitat suitable to lupine could double the next 50 years due to climate change, its role in altering competitive interactions and facilitating succession implies that it may further accelerate climate change-related ecological transition in cold biomes (cf. Hiltbrunner et al., 2014).

The studies discussed so far addressed shifts in baseline (average) temperatures, yet climate warming also leads to a higher incidence of unusual short-term warm spells. Bokhorst et al. focused on warm episodes in winter, which can cause shallow snow packs to melt, subsequently exposing the otherwise sheltered (insulated) vegetation to potentially harmful low temperatures (Treharne et al., 2019). In their experimental study in northern Norway, Bokhorst et al. found that defoliation due to a larval outbreak likely obscured potential winter warming effects, suggesting that impacts in this case were limited to the event exerting the strongest pressure. On the other hand, De Boeck et al. found that two pressures worked synergistically in an alpine grassland. While the impact of a summertime heat wave or drought was insignificant or moderate, respectively, strong impacts were observed when heat and drought coincided (as is often the case naturally), both in the short (De Boeck et al., 2016) and longer term (De Boeck et al.). The finding that a climate extreme led to legacy effects regarding plant cover and community composition years after the original event, highlights that climate change can cause significant impacts in cold biomes other than through long-term changes in mean conditions.

The interactions between warming and changes in precipitation were further explored by Li et al. Their experimental study on the Qinghai–Tibetan Plateau showed that responses to warming very much depended on concomitant changes in precipitation: drier conditions led to a general deterioration of the grassland (especially due to lower productivity), while wetter conditions increased biomass production while preserving forage quality. It is thus critical for sustainable management of these systems that local precipitation trends and variability are taken into consideration in decision-making (e.g., setting maximum grazing intensities). In cold biomes, snowmelt timing is highly important for the onset of the growing season (Klein et al., 2016). Winkler et al. showed that spatial variation in composition and peak cover of an alpine grassland was driven by the timing of snowmelt and the associated dry-down during summer. Some regions may face lower winter snowfall under climate change (Gobiet et al., 2014), but at high latitudes, the opposite is likely, at least in the near future (Christensen et al., 2013). This could affect arctic communities both through increased mineralization rates (Schimel et al., 2004) and changes in the onset of the growing season (Wipf and Rixen, 2010). D'Imperio et al. looked into effects of both increased winter snow amounts and higher summertime temperatures, focusing on root dynamics, which are critically important in these nutrient poor environments. They found that increased winter snowfall and summer warming elicited opposing effects belowground, with the former suppressing and the latter stimulating root growth in this arctic wetland. Because alpine and arctic soils hold important carbon stocks (Hugelius et al., 2014), improving our knowledge on belowground dynamics is important in determining changes in the carbon balance.

To elucidate these and other climate change related issues, long term studies are essential (Knapp et al., 2012). The final contribution to our research topic has done exactly that. Angulo et al. report on an experiment that went on for 10 years in the Spanish Pyrenees and in which *Pinus uncinata* seedlings (later saplings) were exposed to warming, one-time NPK fertilizer addition and/or interaction with the dominant shrub *Rhododendron ferrugineum*. Interestingly, they find that the nature of interactions with this shrub changed as the experiment went on, switching from facilitation by the shrub to competition and ultimately competitive release. Moreover, the impacts of warming and fertilization (including their interactions) differed depending on presence or absence of *R. ferrugineum*, highlighting the importance of considering species interactions in studying global change impacts.

This research topic has shown that the speed with which climate change is happening, encompassing long-term warming but also short-term temperature anomalies and concurrent changes in precipitation, may overwhelm some species and alter community composition and functioning also in the resilient ecosystems of the cold biomes. Persistence in a community will likely depend on the plasticity to cope with both changes in the abiotic and the biotic environment, including interactions with novel (invasive) species. Short and longer term responses may differ, which is why more experiments are needed that monitor communities for multiple years. This is challenging in these often remote locations, and made more difficult as the baseline climate is shifting so rapidly. Understanding, disentangling and predicting the longer-term outcomes of global change impacts in cold biomes will therefore likely remain a frontier in plant ecology.

## Author Contributions

HD wrote the first draft of the editorial, with all co-authors jointly editing the final version.

### Conflict of Interest Statement

The authors declare that the research was conducted in the absence of any commercial or financial relationships that could be construed as a potential conflict of interest.
